# Glucose variability and periodontal disease in type 1 diabetes: a cross-sectional study—The “PAROdontopatia e DIAbete” (PARODIA) project

**DOI:** 10.1007/s00592-021-01720-y

**Published:** 2021-05-17

**Authors:** Ilaria Dicembrini, Luigi Barbato, Lapo Serni, Mariasmeralda Caliri, Laura Pala, Francesco Cairo, Edoardo Mannucci

**Affiliations:** 1 Department of Experimental and Clinical Biomedical Sciences, University of Florence, viale Morgagni 50, 50134 Florence, Italy; 2 Diabetes Unit, Careggi Teaching Hospital, Largo Brambilla 3, 50127 Florence, Italy; 3 Research Unit in Periodontology and Periodontal Medicine, University of Florence, Florence, Italy

**Keywords:** Peridontal disease, Type 1 diabetes, Glucose monitoring, Glucose variability, Glucose coeffeicient of variation

## Abstract

**Aims:**

Periodontal disease (PD) is a chronic inflammation of periodontal tissue associated with infection from specific anaerobic pathogens contained in dental plaque. Both type 1 and type 2 diabetes are associated with an increased prevalence of PDs. A two-way relationship between diabetes and periodontitis has been proposed, with diabetes increasing the risk for periodontitis, and periodontal inflammation negatively affecting glycaemic control. To date, the relationship between PD and glucose variability in type 1 diabetes has not been evaluated. To investigate the prevalence of PD in patients with type 1 diabetes and its association with glycemic control and glucose variability.

**Methods:**

In this cross-sectional study, all enrolled patients were scheduled to attend both a diabetologic and a periodontal visit. HbA1c, glucose coefficient of variation (CV), loss of clinical attachment (CAL), and periodontal probing depth (PPD) were collected.

**Results:**

136 patients were included in the analysis. The prevalence of PD was 63%. A significant correlation was found between mean CAL and glucose CV (*r* = 0.31, *p* = 0.002), but not with HbA1c. Mean PPD was also associated with glucose CV (*r* = 0.27 and 0.044), but not with HbA1c. In a multiple linear regression model, with mean CAL as dependent variable, age, glucose CV, and smoking habit resulted significantly associated (*r* = 0.23, *p* = 0.013; *r* = 0.33, *p* = 0.001; *r* = 0.34, *p* < 0.001, respectively). Assuming mean PPD as dependent variable, multiple linear regression analysis showed a significant association with glucose CV and smoking habits only.

**Conclusions:**

PD is associated with glucose variability in patients with type 1 diabetes also after adjusting for the main confounders.

## Introduction

Periodontal disease (PD) is a chronic inflammation of periodontal tissue associated with infection from specific anaerobic pathogens contained in dental plaque [1]. The progressive destruction of connective tissue attachment and bone support can result in tooth loss and systemic inflammation [2]. PD affects over 30% of adults with severe forms in more than 10% of the population [3].

Both type 1 and type 2 diabetes are associated with an increased prevalence of PD [4, 5]. In addition, among patients with diabetes, a higher HbA1c level is associated with a higher prevalence and severity of PD [6]. It is conceivable that hyperglycemia, through the activation of oxidative stress pathway, induces periodontal damage by exerting detrimental effects in the periodontium as in several other well-known organs [7]. It is also possible that the systemic inflammation determined by PD alters glucose control [8]. Systemic and local changes of pro-inflammatory cytokine levels (i.e., IL-1β, receptor activator of nuclear factor-Kappa B ligand/osteoprotegerin, and IL-6) are noted in both type 1 and type 2 diabetic individuals [9].

The reduction of glucose variability is emerging as a primary goal in the treatment of type 1 diabetes [10]. The relationship between PD and glucose variability has not been extensively studied so far. Aim of the present study is the assessment of the correlation between glucose variability and periodontal disease in type 1 diabetes.

## Material and Methods

The PARODIA Project is a single-centre, cross-sectional study aimed at investigating the prevalence of periodontal disease (PD) in patients with type 1 diabetes and its association with glycemic control and glucose variability.

Type 1 diabetic patients aged ≥ 18 years currently treated with multiple daily insulin injections or continuous subcutaneous insulin infusion, who provided their written informed consent, were enrolled if they had been continuously using for the last three months the FreeStyle Libre Flash Glucose Monitoring (FGM) system (Abbott Diabetes Care, IL), which is fully reimbursed for type 1 diabetes in Tuscany, and the most widely used system for interstitial glucose monitoring in those patients. Subjects with a history of cancer, HIV, bone metabolic disease, history of radiation, or immunosuppressive/modulating therapy were excluded, as well as those who had taken antibiotics, corticosteroids, or non-steroidal anti-inflammatory drugs in the previous 3 months, in order to avoid the interference of confounders.

All patients underwent a standardized measurement of HbA1c (HPLC-assay, Menarini Diagnostic, Florence, Italy). At the Adult Diabetes Clinic in Florence, screening for chronic diabetes-related complications, lipid profile assay, weight, and blood pressure measurements were performed at enrolment, unless available in the last 12 months. FGM data were downloaded using the cloud-based Libreview® system and used for the calculation of glucose coefficient of variation (CV), percentage time with glucose in target range (TIR), in hyperglycemia (> 10 mmol/L) and hypoglycemia (< 3.9 mmol/L).

Clinical evaluation of periodontal disease was done according to international standards [11]. A full-mouth periodontal examination was performed at the Research Unit in Periodontology and Periodontal Medicine, by a single operator using a NCP15 periodontal probe, collecting data (six sites/tooth of each subject) on periodontal probing depths (PPD, as the distance between the gingival margin and the apical limit of gingival crevice) and gingival recession (REC, as the distance between the cemento-enamel junction and the gingival margin). The amount of loss clinical attachment (CAL) for each assessed site was estimated as PPD + REC. Periodontitis case definition system was used to differentiate stage I to IV periodontitis [12].

The primary endpoint was the correlation of severity of PD in patients with type 1 diabetes, as assessed through CAL, with glucose variability, expressed by glucose CV. Assuming an estimated PD prevalence of 25% [13] and with a precision of 7%, the projected sample size was of 150 patients. Descriptive statistics was performed calculating mean ± Standard Deviation (SD) and median [quartiles], depending on data distribution. Associations between age, sex, HbA1c, diabetes duration, glucose CV, mean CAL, and smoking habits were explored calculating Spearman’s correlations. Linear regression models were applied for multivariate analyses.

In the multiple linear regression analysis, mean CAL or glucose CV, respectively, will be considered as the dependent variable, whereas age, glucose CV, or mean CAL and smoking habit as the independent ones. All analyses were performed using SPSS 26.

The study methodology was designed in guidance by STROBE guideline for cross-sectional study.

## Results

Of the 150 enrolled patients, 136 attended the scheduled periodontal visit and were therefore included in analysis. The characteristics were not significantly different from those of the enrolled population (Table 1). The prevalence of PD, according to the diagnostic criteria specified above, was 63% (stage I n = 14; stage II n = 20; stage III n = 43; Stage IV n = 9). Periodontal parameters are reported in Table 1.

**Table 1:** Characteristics of all enrolled patients and completers

Characteristics	Enrolled	Completers
n	150	136
Male [n (%)]	72(48)	60(44)
Female [n (%)]	78(52)	76 (56)
Age (years)	45 [19–81]	45 [19–81]
Smoker (n)	26	23
No smoker (n)	124	113
Diabetes duration (years)	19 [1–59]	19 [1–59]
BMI (kg/m^2^)	24.0±4.8	24.8±4.8
Total cholesterol (mg/dl)	184±31	187±31
HDL (mg/dl)	62±15	63±15
Triglyceride (mg/dl)	73±31	74±32
Systolic blood pressure (mmHg)	124±12	124±13
Diastolic blood pressure (mmHg)	73±8	73±9
Insulin pump users [n (%)]	46 (30)	44 (32)
Patient with diabetic complications [n (%)]	50 (33)	47 (34.5)
Cardiovascular	4	4
Peripheral arteriopathy	4	4
Retinopathy		
NPDR	37	32
PDR	11	8
Neuropathy	14	14
Renal		
Microalbuminuria	7	7
Macroalbuminuria	0	0
Reduction eGFR	1	1
Glicemic control		
HbA1c (mmol/mol)	58±15	58±15
Average glucose level (mmol/L)	8.9±1.8	9.0±1.8
Glucose coefficient of variation (%)	39±8	39±8
Time in range (%)	59.5±15	59±16
Time in hypoglycemia (%)	7±6	7±6
Time in hyperglycemia (%)	33.5±17	34±18
Periodontal parameters		
Mean PD (mm)	2.71±0.62	2.70±0.61
Mean CAL (mm)	3.01±0.90	3.02±0.91

Values are presented as mean ± Standard Deviation (SD) except for age and diabetes duration expressed as median [range]. NPDR, not proliferative diabetic retinopathy; PDR, proliferative diabetic retinopathy. Cardiovascular complications: Myocardial infarction, stroke, and/or prior coronary revascularization. Neuropathy includes sensitive, motor-sensitive, autonomic neuropathies. PD: periodontal probing depths (as the distance between the gingival margin and the apical limit of gingival crevice). CAL: loss of clinical attachment

**Fig. 1:**
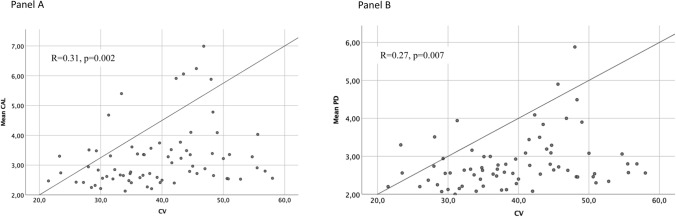
**a** Correlation between the mean amount of loss clinical attachment mean (mean CAL; mm) and glucose coefficient of variation (CV;%). **b** Correlation between mean periodontal probing depth (mean PPD; mm) and glucose coefficient of variation (CV;%)

A significant correlation was found between mean CAL and glucose CV (*r* = 0.31, *p* = 0.002; Fig. 1 Panel A), but not HbA1c (*r* = 0.038 *p* = 0.673). Mean PPD was also associated with glucose CV, but not with HbA1c (*r* = 0.27 and 0.044; *p* = 0.007 and 0.619, respectively; Fig. 1 Panel B). In a multiple linear regression model, with mean CAL as dependent variable, age, glucose CV, and smoking habit resulted significantly associated (*r* = 0.23, *p* = 0.013; *r* = 0.33, *p* = 0.001; *r* = 0.34, *p* < 0.001, respectively). Assuming mean PPD as dependent variable, multiple linear regression analysis showed a significant association with glucose CV and smoking habits only (*r* = 0.23, *p* = 0.019; *r* = 0.33, *p* = 0.001, respectively).

An alternative model, with glucose CV as dependent variable, showed a significant association of mean CAL with glucose CV, even after adjusting for age and smoking habits (*r* = 0.29, *p* = 0.013) and significant association of mean PPD with glucose, even after adjusting for smoking habits (*r* = 0.26, *p* = 0.029). No correlation was found with TIR, time in hypoglycemia and in hyperglycemia (data not shown).

## Discussion

The prevalence of PD in this study was higher than that reported in recently published meta-analysis of cross-sectional studies [4]. This difference could be due, at least partly, to differences in assessment methods, diagnostic, and classification criteria. Notably, studies with more accurate assessment methods, similar to those used in the present survey, provided higher prevalence estimates [14, 15]. However, differences across genetically, geographically, and culturally heterogeneous populations cannot be ruled out.

A higher glucose variability, estimated as glucose CV, was associated with both mean CAL (which is an expression of PD-related bone loss) and mean PPD (which indicates local inflammation).

To our knowledge, this is the first available report of such an association. Those associations were confirmed at multivariate analyses after adjusting for the main confounders, i.e., smoking and age. Conversely, no significant association of either mean PPD or mean CAL was observed with HbA1c. Although this latter result may seem at variance with prior cross-sectional studies [4], it may be argued that the mean and the variance of HbA1c in the present sample were relatively low, possibly attenuating existing correlations.

A cross-sectional study does not allow causal inferences. Glucose fluctuations could facilitate the development of PD, and PD-induced systemic inflammation could affect glucose control [16]. Notably, in multivariate analyses the association of mean CAL and glucose CV was significant in models in which either mean CAL or glucose CV were imputed as dependent variable, supporting the possibility of a bi-directional association [16]. Causal relationships can be explored only through interventional studies. In one small trial in type 2 diabetes, the improvement of glycemic control produced marginal benefits on PD [17]. On the other hand, a Cochrane Review on the treatment of PD in mostly type 2 diabetic patients, including 35 randomized trials, showed a low-quality evidence of a modest short-term improvement of HbA1c [5].

No specific data on the effect of PD treatment on glucose variability have been reported so far.

Some limitations of the present study should be recognized. The sample size, although deemed sufficient for the principal endpoint with a formal power calculation, is relatively small. In addition, the sample cannot be considered representative of the whole population of patients with type 1 diabetes. On the other hand, the study has some strengths, such as the accuracy in the assessment of PD, and the availability of glucose monitoring data from all patients.

In conclusion, periodontal disease is associated with glucose variability in patients with type 1 diabetes. Further, interventional studies are needed to verify the effect of reduction of glucose variability on the evolution of periodontal disease, and those of periodontal treatment on glucose variability.
